# Immunoproteasome Deficiency Modifies the Alternative Pathway of NFκB Signaling

**DOI:** 10.1371/journal.pone.0056187

**Published:** 2013-02-14

**Authors:** Marcela Maldonado, Rebecca J. Kapphahn, Marcia R. Terluk, Neal D. Heuss, Ching Yuan, Dale S. Gregerson, Deborah A. Ferrington

**Affiliations:** Department of Ophthalmology and Visual Neurosciences, University of Minnesota, Minneapolis, Minnesota, United States of America; Johns Hopkins School of Medicine, United States of America

## Abstract

Immunoproteasome is a protease abundant in immune cells and also present, albeit at lower concentrations, in cells outside the immune system. Recent evidence supports a novel role for the immunoproteasome in the cellular stress response potentially through regulation of NFκB signaling, which is the primary response to multiple stressors. The current study tests whether the Classical or Alternative Pathways are regulated by immunoproteasome following chronic TNFα exposure in cultured retinal pigment epithelial cells isolated from wild-type mice and mice deficient in one (LMP2, L2) or two (LMP7 and MECL-1, L7M1) immunoproteasome subunits. Assays were performed to assess the expression of NFκB responsive genes, the content and activity of NFκB transcription factors (p65, p50, p52, cRel, RelB), and expression and content of regulatory proteins (IκBα, A20, RPS3). Major findings include distinct differences in expression of NFκB responsive genes in both KO cells. The mechanism responsible for the altered gene expression could not be established for L7M1 since no major differences in NFκB transcription factor content or activation were observed. However, L2 cells exhibited substantially higher content and diminished activation of NFκB transcription factors associated with the Alternative Pathway and delayed termination of the Classical Pathway. These results provide strong experimental evidence supporting a role for immunoproteasome in modulating NFκB signaling.

## Introduction

The proteasome is a proteolytic complex that regulates cellular processes essential for cell survival, such as cell cycle, signal transduction, gene expression, and degradation of damaged and misfolded proteins. Several proteasome subtypes, defined by their catalytic subunits, have been described [1], [2]. In the standard proteasome, the catalytic subunits are β1, β2, and β5. These subunits cleave proteins after acidic, basic, and hydrophobic amino acids, respectively. In nascent proteasome cores, the standard catalytic subunits can be replaced by the inducible subunits, LMP2 (β1i), MECL-1 (β2i) and LMP7 (β5i) to form the catalytic core of the immunoproteasome (i-proteasome). While there are minor differences in the catalytic activities of the β2/MECL and β5/LMP7 subunits for standard and i-proteasome, the activity of β1 and LMP2 differ. LMP2 preferentially cleaves after hydrophobic amino acids rather than after acidic amino acids, generating a population of peptides that are enriched in hydrophobic C-termini. This difference in cleavage specificity is important for i-proteasome's role in immune function. Intermediate cores containing a mixture of standard and inducible catalytic subunits have also been reported [3].

Proteasome subtypes differ substantially not only in their enzymatic characteristics but also in their pattern of expression, suggesting the potential for discrete contributions to cell processes. Standard proteasomes are constitutively expressed in nearly all mammalian cells. In contrast, i-proteasome is highly expressed in cells of the immune system, where it performs functions associated with generating peptide ligands for MHC class I antigen presentation [4]. I-proteasome is also present, albeit in low abundance under basal conditions, in cells outside the immune system, including neurons of the retina and brain, skeletal muscle and epithelial cells of the retina [5]–7. When these cells are exposed to various stressors, such as inflammatory cytokines, disease, or oxidative stress, i-proteasome is significantly upregulated [8]–[11]. In addition to its rapid induction, assembly of the nascent i-proteasome core particle is four times faster than the standard core and conversely, i-proteasome's half-life is substantially shorter [12]. This highly dynamic adjustment in i-proteasome content permits its rapid response to environmental challenges. Taken together, these results suggest a role for i-proteasome in regulating processes associated with the cellular response to stress and injury.

The nuclear factor-kappa B (NFκB) pathway is the primary mechanism for responding to multiple stressors, such as toxic chemicals, UV light, and oxidative damage, as well as pro-inflammatory cytokines, viral and bacterial products. Activation of the NFκB pathway elicits rapid induction of early response genes that help protect the cell from damage. However, aberrant regulation or long-term activation of NFκB signaling can lead to pathologies, such as toxic shock, neurodegenerative and inflammatory diseases [13].

The genes *rela, relb, crel, nfκb1*, and *nfκb2* encode the five mammalian NFκB transcription factors, RelA (p65), RelB, c-Rel, p105/p50, and p100/p52, respectively. These proteins can associate to form homo- and heterodimers that translocate into the nucleus and bind to the κB element of target genes. Only RelA (p65), c-Rel and RelB have the transcription activation domain required for inducing gene expression. Consequently, three transcriptionally inactive dimers (p50/p50, p52/p50, and p52/p52) actually inhibit transcription by blocking the promoter region [14].

Prior to stimulation, NFκB dimers are sequestered in the cytoplasm by inhibitory proteins, including the IκBs (α, β, ε) and the precursors of active transcription factors, p105 and p100. Upon stimulation of NFκB through either binding of a ligand to its cognate receptor or through a receptor independent event (i.e., oxidative stress), a cascade involving activation of multiple kinases and phosphorylation of select proteins in the NFκB pathway is initiated. Proteasome plays a direct role in regulating NFκB via complete degradation of the inhibitor IκB, releasing the active dimer and allowing its translocation into the nucleus. This pathway, typically involving p65/p50 dimer, is referred to as the Classical Pathway. A second mechanism involves endoproteolysis or partial degradation of the inhibitory portion of p105 and p100, generating the active transcription factors p50 and p52. In addition to p65, these transcription factors can associate with RelB or co-associate to form homo and heterodimers. This pathway is referred to as the Alternative Pathway. While initially believed to be functionally separate, more recent evidence suggests considerable crosstalk between the Classical and Alternative NFκB pathways [15]. Crosstalk between the two pathways has been well established in immune cells following TNFα-stimulation and is an integral part of the innate and adaptive immune response [16].

An important component of regulation built into the NFκB pathway is the induction of multiple inhibitors that serve to attenuate or terminate the signal. These inhibitors include the IκBs, p100, and p105, which sequester transcription factor dimers in the cytoplasm. Additionally, IκBα can terminate the signal by removing the p50/p65 dimer from the promoter. Specific to terminating TNFα-induced stimulation of NFκB is the inhibitor A20 (Fig. S1). This protein is an E3 ligase that ubiquitinates RIP1, a modular protein located at the TNF receptor, and targets it for proteasome degradation. The absence of RIP1 terminates NFκB signaling at the receptor [17].

Evidence in both KO mice and humans with i-proteasome mutations implicate a role for the i-proteasome in modulating NFκB signaling. In i-proteasome-deficient mice, defects in proteolytic processing of NFκB precursors (p100/p105) and decreased degradation of IκBα have been reported [18]–[20]. While these initial findings were later disputed [21]–[22], more recent reports in patients with mutations in the LMP7 protein [23] have re-energized this controversy. Notably, patients harboring mutations in LMP7 consequently have lower i-proteasome content and exhibit a spectrum of auto-inflammatory syndromes (i.e., higher IL-6 levels) that implicate aberrant NFκB signaling as part of the disease mechanism [23].

The current study tests the hypothesis that i-proteasome modulates NFκB signaling by comparing the early and late response to chronic TNFα exposure in cultured retinal pigment epithelial (RPE) cells isolated from wild-type mice and mice deficient in one (LMP2, referred to as L2) or two (LMP7 and MECL-1, referred to as L7M1) subunits of the i-proteasome. TNFα was used as the stimulant because (1) the mechanism of TNFα-induced NFκB signaling is well-described (Fig. S1), and (2) chronic stimulation with TNFα provides a means for generating an NFκB response similar to that observed with chronic inflammation. Cell lines deficient in i-proteasome subunits may provide important mechanistic details about NFκB signaling that are relevant to the chronic inflammation experienced by patients with mutations in i-proteasome genes. Cultured cells from the retinal pigment epithelium were used because they express i-proteasome in measurable amounts under basal conditions and can strongly upregulate i-proteasome under conditions of stress or inflammation [8], [10]. These cells are responsive to TNFα and have an immunomodulatory role in vivo, as demonstrated by their secretion of the pro-inflammatory molecule IL-6 and their ability to induce unresponsiveness in T-cells [24]. The current study tests whether RPE behave similar to immune cells in activating both the Classical and Alternative Pathways in response to TNFα [16]. The use of cells from KO mice allowed us to determine if altering the i-proteasome content affects the TNFα response. Results from the current study revealed distinct differences in expression of NFκB responsive genes in cells lacking either one (L2) or two (L7M1) i-proteasome subunits. Our data showed higher content and diminished activation of NFκB transcription factors associated with the Alternative Pathway and delayed termination of the Classical Pathway in cells deficient in the LMP2 subunit.

## Materials and Methods

### Cell culture conditions

Retinal pigment epithelial (RPE) cells isolated from wild-type (WT) and knock-out (KO) mice deficient in one (LMP2, referred to as L2) or two (LMP7 and MECL-1, referred to as L7M1) subunits of the i-proteasome were immortalized as previously described [25]. This study includes results from two independently derived cell lines for WT and KO mice. An animal protocol for harvesting RPE was approved by the Institutional Animal Care and Use Committee of the University of Minnesota and followed the guidelines established by the National Institutes of Health. RPE cells were cultured in growth media containing Dulbecco's Modified Eagle Medium, 0.4 mM L-glutamine, 25 mM glucose (Gibco), MEM non-essential amino acids (Cellgro), 50 U/mL penicillin, 50 U/mL streptomycin (Gibco) and 4% heat inactivated fetal bovine serum (Atlanta Biologicals). Cells were cultured at 37°C in a humidified chamber containing 5% C0_2_. For TNFα treatments, cells were treated with recombinant human TNFα (10 ng/mL, R&D Systems) and harvested at times indicated in figures.

### Preparation of tissue homogenates

In order to harvest murine RPE cells, the anterior segment was removed from enucleated globes. The retina was then removed and RPE cells were collected by gentle agitation in 1X PBS with a fine paint brush. The cells were pelleted by centrifugation at 600×g for 10 min. Whole cell lysates were prepared after two cycles of freeze thaw with liquid nitrogen, followed by homogenization with a glass dounce homogenizer using a buffer containing 50 mM Tris-HCl pH 7.8, 2% CHAPS.

Cerebellar tissue was dissected away from the brain stem after brains were removed from the skull. Tissue homogenates were prepared using a glass dounce homogenizer with a buffer containing 50 mM Tris-HCl pH 7.8, 2% CHAPS. Protein concentrations were determined using the bicinchoninic acid (BCA) assay (Pierce, Rockford IL) with bovine serum albumin as the standard.

### Proteasome Activity

The fluorogenic peptides LLE-AMC (200 µM), LLVY-AMC (75 µM) (EMD Biosciences, San Diego, CA), and VGR-AMC (150 µM) (Biomol, Plymouth Meeting, PA), were used to measure the caspase-like, chymotrypsin-like and trypsin-like activities, respectively, as outlined [26]. Assays were done in the presence or absence of the proteasome inhibitor MG132 (200 µM) or Lactacystin (50 µM). Hydrolysis of the fluorogenic peptides was measured in buffer containing 50 mM Tris (pH 7.5), 5 mM MgCl_2_, 20 mM KCl and 0.5 mM ATP.

### IL-6 ELISA

Cells were grown in 96-well culture plates. Cell culture media was harvested from untreated cells or at 48 hrs after a single dose of TNFα (10 ng/mL)(1×TNF). In a second experiment, cells were treated with TNFα (10 ng/mL) for 48 hrs, media was removed and replaced with either fresh media (wash-out) or media containing 10 ng/mL TNFα (2×TNF). Cell media was harvested at 96 hrs after the initial treatment. The IL-6 content of culture media was determined by ELISA, using anti-mouse IL-6 (eBioscience, clone MP5-20F3) for the capture antibody, biotin-conjugated anti-mouse IL-6 (eBioscience, clone MP5-32C11) for the detection antibody, and the enzyme-conjugated secondary antibody, AKP-Streptavidin (BD Pharmingen). A standard curve of recombinant mouse IL-6 (eBioscience) was used to calculate the amount of secreted IL-6. The content of secreted IL-6 was normalized to cell density for each well using the CyQuant NF cell proliferation Assay (Invitrogen). IL-6 production is reported as picograms IL-6 per cell (pg/cell).

### Isolation of Nuclear/Cytoplamic Fractions and whole cell lysates

Nuclear and cytoplasmic fractions were prepared immediately from cell pellets using NE-PER Nuclear and Cytoplasmic Extraction Reagents kit (Pierce, Rockford IL). Fractions containing either cytoplasmic or nuclear proteins were stored at −80°C. Whole cell lysates were prepared by lysing cells in buffer containing 10 mM KCl, 25 mM Tris, pH 7.8, 2.5 mM EDTA, 0.5% NP40, 10% glycerol. Lysates were cleared by centrifugation at 600×g for 15 minutes. Cell pellets used to generate whole cell lysates were stored at −80°C until use. Protein concentrations were determined using bicinchoninic acid (BCA) assay (Pierce, Rockford IL) with bovine serum albumin as the standard.

### Western blot analysis

Proteins were electrophoretically separated on SDS-polyacrylamide gels (10% or 13%) and transferred to either nitrocellulose (for NFκB antibodies) or PVDF membranes (all other antibodies), as previously described [25]. The linear range of detection was determined for each antibody from blots containing incremental increases in protein. Loading controls were GAPDH, Beta actin, or histone H2B (nuclear fraction). Information for primary antibodies, linear range, dilutions, and protein loads for each antibody are provided in Table 1. Images were captured using a ChemiDoc XRS (Bio-Rad Laboratories, Hercules, CA) and analyzed using Quantity One (Bio-Rad Laboratories). The density of sample immune reactions was normalized to a WT control (untreated cells) run on each blot. For quantification of proteasome subunits, a sample of 20S purified from liver was run on each blot to ensure that the immune reactions were from subunits incorporated into the core.

**Table 1:** Antibodies used for Western blotting.

Antibody	Type	Linear Range (µg)	Protein Load (µg)	Company
p100/p52	R	2–30	25	Cell Signaling Technology, Danvers, MA
p105/p50	R	2–30	25	Epitomics, Burlingame, CA
IκBα	M	2–20	10	Cell Signaling Technology, Danvers, MA
Ribosomal protein S3 (RPS3)	R	5–30	25	Cell Signaling Technology, Danvers, MA
A20	R	5–25	10	Cell Signaling Technology, Danvers, MA
RelB	R	5–25	25	Cell Signaling Technology, Danvers, MA
p65	R	5–70	10	Cell Signaling Technology, Danvers, MA
Phospho-p65 (Ser 536)	R			Abcam, Cambridge, MA
Proteasome subunit α7	M	2–10	5	Enzo Life Sciences, Farmingdale, NY
Proteasome subunit 20SX (β5)	R	2–10	5	Thermo Scientific, Rockford, IL
Proteasome subunit 20SY (β1)	R	2–10	5	Thermo Scientific, Rockford, IL
Proteasome subunit LMP2 (β1i)	M	2–35	15	Enzo Life Sciences, Farmingdale, NY
Proteasome subunit LMP7 (β5i)	R	2–35	15	Enzo Life Sciences, Farmingdale, NY
RPE65	M		50	Abcam, Cambridge, MA
Beta Actin	M			Santa Cruz Biotechnology, Santa Cruz, CA
GAPDH	M			Meridian Life Sciences, Memphis, TN
Histone H2B	R			EMD Millipore, Billerica, MA

All antibodies were isotype IgG and were diluted to 1∶1000.

Monoclonal, host species mouse (M); polyclonal, host species rabbit (R).

### Phagocytosis measured by flow cytometry

Cells were cultured in 6-well plates. Growth medium was removed from confluent cells and replaced with fresh growth medium containing 1.0 µm polystyrene beads loaded with yellow-green fluorescent dye (FluoSpheres, Molecular Probes; Eugene, OR) at a concentration of 1.4×10^5^ microspheres/mL. RPE cells were incubated with the beads for 24 hrs to allow for phagocytosis, then washed 2× and harvested by trypsinization. The cells were prepared for flow cytometry in growth medium to ensure the removal of beads outside the RPE. Cell pellets were resuspended in PBS containing 2 mM EDTA and 0.5% BSA. The resulting cell suspensions was used to determine microbead content (ex/em = 505 nm/515 nm) per cell using flow cytometry (FACSCalibur with Cell Quest software, BD Biosciences) and analyzed using FlowJo software (Tree Star, Ashland, OR).

### RNA isolation

Total mRNA was isolated from cells pellets using RNeasy Mini kit (Qiagen; Valencia, CA). cDNA was generated in a GeneAmp PCR System 9700 (Applied Biosystems, Forester City, CA) from RNA (70 ng) using SuperScript III reverse transcriptase (Invitrogen) and oligo-dTs (Integrated DNA technologies).

### Quantitative RT-PCR

Quantitative real-time PCR was performed using an iQ5 Multicolor Real-time PCR I-cycler (Bio-Rad Laboratories, Hercules,CA). Reactions were performed with cDNA (2 ng/reaction, performed in triplicate) using Immolase DNA polymerase (Bioline), 800 µM dNTP mix (Bioline), SYBR Green (Invitrogen), and 200 nM of forward and reverse primers. Normalized gene expression was determined using the iQ5 optical system software (Bio-Rad Laboratories, Hercules,CA) using acidic ribosomal phosphoprotein P0 (ARBP) expression as a reference gene for each sample. The sequence for primers used in qRT-PCR is provided in Table 2.

**Table 2:** Primers used for Real-Time RT-PCR.

Gene	Product (bp)	Primer Sequence-Forward	Primer Sequence-Reverse
*arbp*	102	5′-CTT TCT GGA GGG TGT CCG CAA-3′	5′-ACG CGC TTG TAC CCA TTG ATGA-3′
*Il-6*	91	5′-GTT GCC TTC TTG GGA CTG ATG-3′	5′-TGG GAG TGG TAT CCT CTG TGA A-3′
*nfκb2*	75	5′-TGG AAC AGC CCA AAC AGC -3′	5′-CAC CTG GCA AAC CTC CAT -3′
*inos*	61	5′-GGC AGC CTG TGA GAC CTT TG -3′	5′-GAA GCG TTT CGG GAT CTG AA -3′
*nfκb1*	90	5′-GAG TAC GAC AAC ATC TCC TTG G -3′	5′-CAG AGG TGT AGT CCC ATC ATA -3′
*a20*	69	5′-GCA AGG CTG GGA CCA CG -3′	5′-TTG GGT AAG TTA GCT TCA TCC AAT T -3′
*relb*	204	5′-GCT ACG GTG TGG ACA AGA AG -3′	5′-TGG AAG CAG GGA AGA AAT CAG -3′
*iκbα*	200	5′-GTC AAC AGG GTA ACC TAC CA -3′	5′-CCT CCA AAC ACA CAG TCA TC -3′
*cox2*	162	5′-CAT GGA CTC ACT CAG TTT GTT -3′	5′-GAA GCG TTT GCG GTA CTC ATT -3′

### Endpoint PCR

Expression of pigment epithelium derived factor (PEDF), bestrophin (BEST1), and beta actin (control) were evaluated as previously described [25]. Primer sequences were as follows: PEDF *For*: GCTTACTTCAAGGGGCAGTG; PEDF *Rev*: ACATTAAGTGCTACTGGGGT. Beta Actin *For*: AGGTGACAGCATTGCTTCTG; Beta Actin *Rev*: GCTGCCTCAACACCTCAAC. Best-1, *Vmd2 For*: ACACAACACATTCTGGGTGC; Best-1/*Vmd2 Rev*: TTCAGAAACTGCTTCCCGATC. PCR amplification of cDNA was performed with the *Taq* polymerase (Invitrogen Life Technologies) using the GeneAmp PCR System 9700 (Applied Biosystems, Forester City, CA). PCR products were separated on a 2% agarose gel and visualized by staining with ethidium bromide.

### NFκB transcription factor activation assay

Activity of individual NFκB transcription factors was determined in nuclear extracts (5 µg) using the ELISA-based TransAM NFκB Transcription Factor assay kit (Active Motif; Carslbad, CA). Experiments were performed on WT, L7M1, and L2 cells cultured and processed in parallel. The assay was performed for three to five separate cell preparations, each measured in duplicate. Data were normalized to WT untreated cells in each experiment.

### Statistical Analysis

Data were analyzed by two-way ANOVA with mouse strain and time as the main effects, using the statistical software NCSS (v. 2001, Kaysville, UT). One-way ANOVA was performed for cells from each mouse strain to determine if there was a significant response to TNFα. Tukey-Kramer post-hoc test was performed to determine differences between groups. Significance was set at p<0.05. Data are reported as mean ± standard error of the mean.

## Results

### Characterization of cells

**Figure 1:**
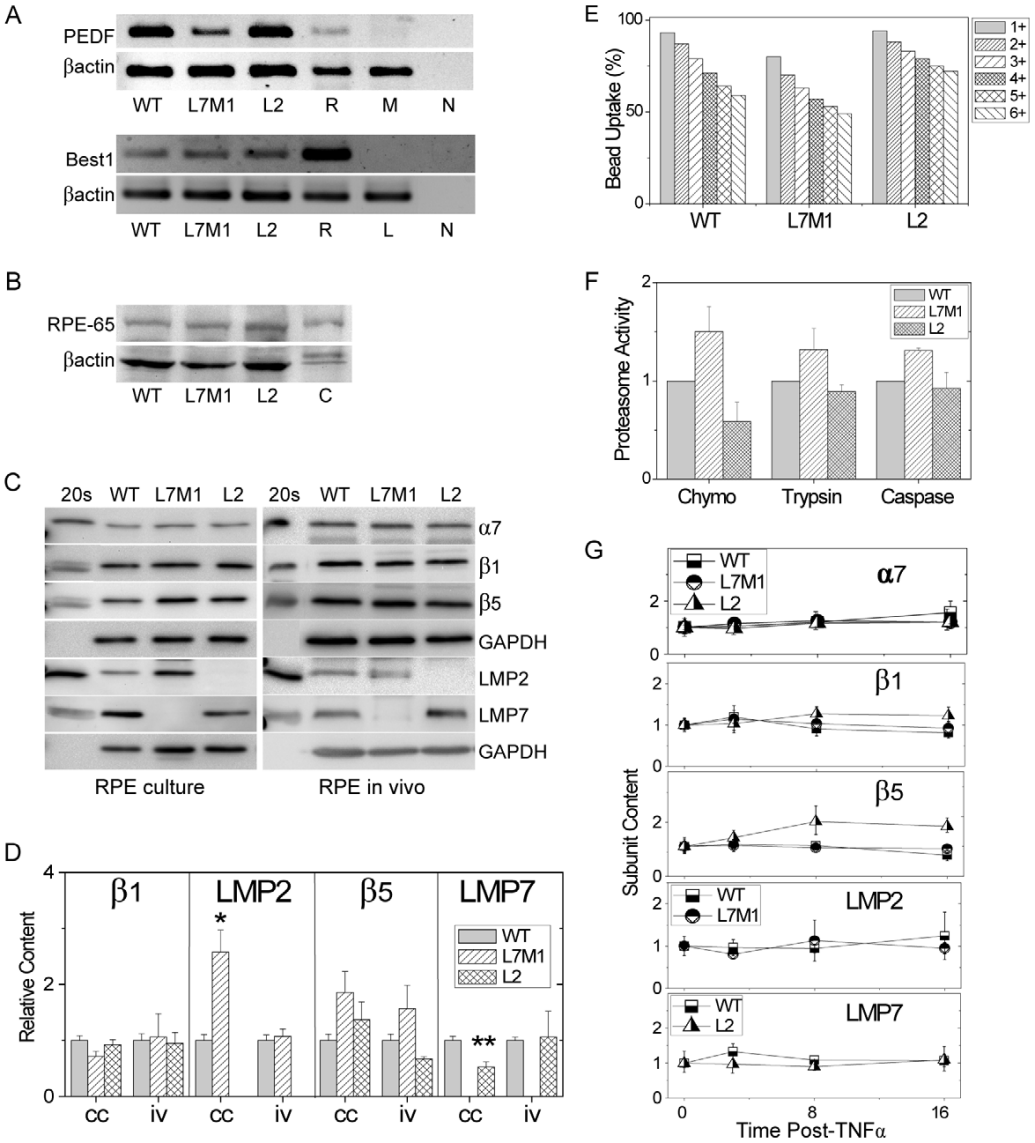
Characterization of Murine RPE Cell Lines. (**A**) Gels show RT-PCR products for pigment epithelium-derived factor (PEDF), bestrophin1 (Best1), and βactin in cultured RPE isolated from WT, L7M1, and L2 mice. (R) mouse RPE (positive control), (M) Muscle, and (L) lens, negative controls, (N) no template control. (**B**) Western blots showing reaction for RPE-65 and βactin in cultured RPE cells (50 µg per lane). (C) Reaction from RPE harvested from mice (positive control). (**C**) Western blots showing reactions for proteasome subunits from cultured cells (RPE culture) and RPE harvested from mice, (RPE in vivo). Protein loads were 5 µg per lane for α7, β1, and β5. Protein loads for LMP2 and LMP7 were 15 µg for cultured RPE and 25 µg for RPE tissue harvested from mice. Glyceraldehyde 3-phosphate dehydrogenase (GAPDH) was used as a loading control. The 20 s reaction was used as a positive control. (**D**)Summary of proteasome subunit content measured from Western blots at baseline (no TNFα) in RPE culture (cc) (n = 2 cell lines/group) and in vivo RPE harvested from mice (iv) (n = 3 mice/group). *p = 0.01, **p = 0.004. (**E**) Flow cytometry of phagocytosed 1 µm YG-labeled latex beads. Bars represent the number of beads internalized by individual cells. Graph summarizes the percent cells containing 1 or more beads. Data shown are representative of two independent experiments. (**F**) Proteasomal catalytic activity in WT and i-proteasome-deficient mice (L7M1 and L2). Hydrolysis of the fluorogenic peptides LLVY-AMC, VGR-AMC, and LLE-AMC measured the chymotrypsin-like (Chymo), trypsin-like (Trypsin), and caspase-like (Caspase) activities, respectively. (**G**) Summary of proteasome subunit content measured from Western blots at baseline (no TNFα) and after TNFα (10 ng/mL) stimulation. Subunit immune reactions were normalized to a standard sample run on each blot and to the total proteasome content for each preparation. The content of each subunit is shown relative to the reaction at baseline. Data shown are the mean (±SEM) of three independent experiments.

To test how the absence of specific i-proteasome subunits affected NFκB signaling, we used two independently-derived cell lines that were generated from RPE cells harvested from WT mice and mice deficient in one (L2) or two (L7M1) i-proteasome subunits, as previously described [25]. All of the cell lines express PEDF, Best1, and RPE65 (Fig. 1A,B), and are able to phagocytose latex beads (Fig. 1E). These results confirm that the cultured cells have retained these in vivo characteristics specific to RPE cells.

Proteasome activity was measured in each cell line using fluorogenic peptide substrates to monitor the caspase-, trypsin-, and chymotrypsin-like activities (Fig. 1F). For L7M1 and L2 cells, the chymotrypsin-like activity was ∼40% higher and lower, respectively. However, these results did not achieve statistical significance (p = 0.09). Trypsin- (p = 0.12) and caspase-like (p = 0.20) activities were also not different between WT and KO cells.

Proteasome subunit composition was evaluated from Western immunoblots using subunit-specific antibodies (Fig. 1C,D). The reaction of the α7 subunit was used to estimate proteasome total content since it is constitutively expressed in all proteasome cores [8]. For the β-subunits, alignment of the immune reaction of the 20S isolated from liver with sample immune reactions was used to ensure that measurements were performed on mature proteins that had incorporated into the 20S core. As expected, there was no immune reaction associated with L7M1 and L2 for the LMP7 and LMP2 subunits, respectively, as predicted from the gene disruption [27]–[29]. Densitometric analysis of the immune reactions for two independently derived RPE cultures showed no significant difference in both the α7 (data not shown) and β1 subunits when comparing WT and KO cells (Fig. 1D). While β5 content was ∼80% higher in L7M1 cells compared to WT, this increase did not reach statistical significance (p = 0.12). For the immunoproteasome subunits, LMP2 was increased more than 2.5-fold in L7M1 cells (p = 0.01) and LMP7 was decreased ∼50% in L2 cells (p = 0.004).

Subunit composition was also measured in RPE cells harvested from murine eyes to determine how closely the cultured cells replicate the in vivo state (Fig. 1C,D). The relative content of α7, β1, LMP2, and LMP7 were not different between WT and KO mice. Similar to cultured cells, there was a 60% increase in content of the β5 subunit in L7M1 RPE (p = 0.11). These results show the main difference between cultured RPE and cells in vivo is the significantly greater content of LMP2 in cultured cells from L7M1 mice.

The high LMP2 content in L7M1 cultures and equivalent incorporation of the LMP2 subunit in L7M1 RPE in vivo are in direct contrast to previous reports from other tissues, which showed that in the absence of LMP7 there was significantly less LMP2 incorporation [30]–[32]. While our previous results for the 20S purified from spleen are consistent with inhibited LMP2 incorporation in L7M1 mice, we have also shown LMP2 incorporation is not inhibited in L7M1 retinas or in RPE cell cultures derived from a different set of KO mice [8]. Taken together, these results suggest there are tissue-specific differences in proteasome subunit composition. To directly test this possibility, we monitored proteasome subunit composition in the cerebellum from WT and KO mice. We have previously shown that within this region of the brain, i-proteasome is expressed in measurable amounts in glia, neurons, oligodendrocytes, and bone marrow-derived immune cells [5]. Under identical experimental conditions used to estimate proteasome subunit composition in RPE cells, we found that LMP2 content was three-fold lower in the cerebellum of L7M1 mice (Supplement Fig. S2). These results suggest that brain and retinal cells in vivo and in cultures have different proteasome populations in L7M1 mice. Furthermore, the LMP7 subunit is not absolutely required for LMP2 incorporation in certain cells. Thus, RPE cells provide a unique experimental setting for testing differences in immunoproteasome content.

To determine the optimal dose of TNFα to achieve NFκB activation, preliminary experiments tested a range of TNFα concentrations (0.5 to 15 ng/ml). Based on the dose-dependent expression of IL-6 and cell viability assays showing that cells remained viable over 48 hrs, a dose of 10 ng/ml TNFα was selected for experiments (data not shown). Western blotting was used to evaluate the proteasome population in each cell line and determine whether the subunit content changed with TNFα treatment over the time course of our experiments. Densitometry of the immune reaction showed no change in proteasome subunits throughout 16 hours of exposure to TNFα (Fig. 1G). These results indicate that the proteasome population remained stable over the time frame of our assays.

### Altered Expression of NFκB responsive genes

An early outcome of NFκB activation is the upregulated expression of multiple genes containing the κB enhancer element in the promoter [14]. However, the magnitude and kinetics of activation depend upon the type of stimuli and cell. The expression of NFκB transcription factors (*nfκb1, nfκb2, relb*), negative regulators (*iκba, a20*), and three prototypic NFκB responsive genes (*inos, cox2, il6*), which are all regulated by NFκB, was measured by quantitative RT-PCR following treatment with TNFα.

**Figure 2:**
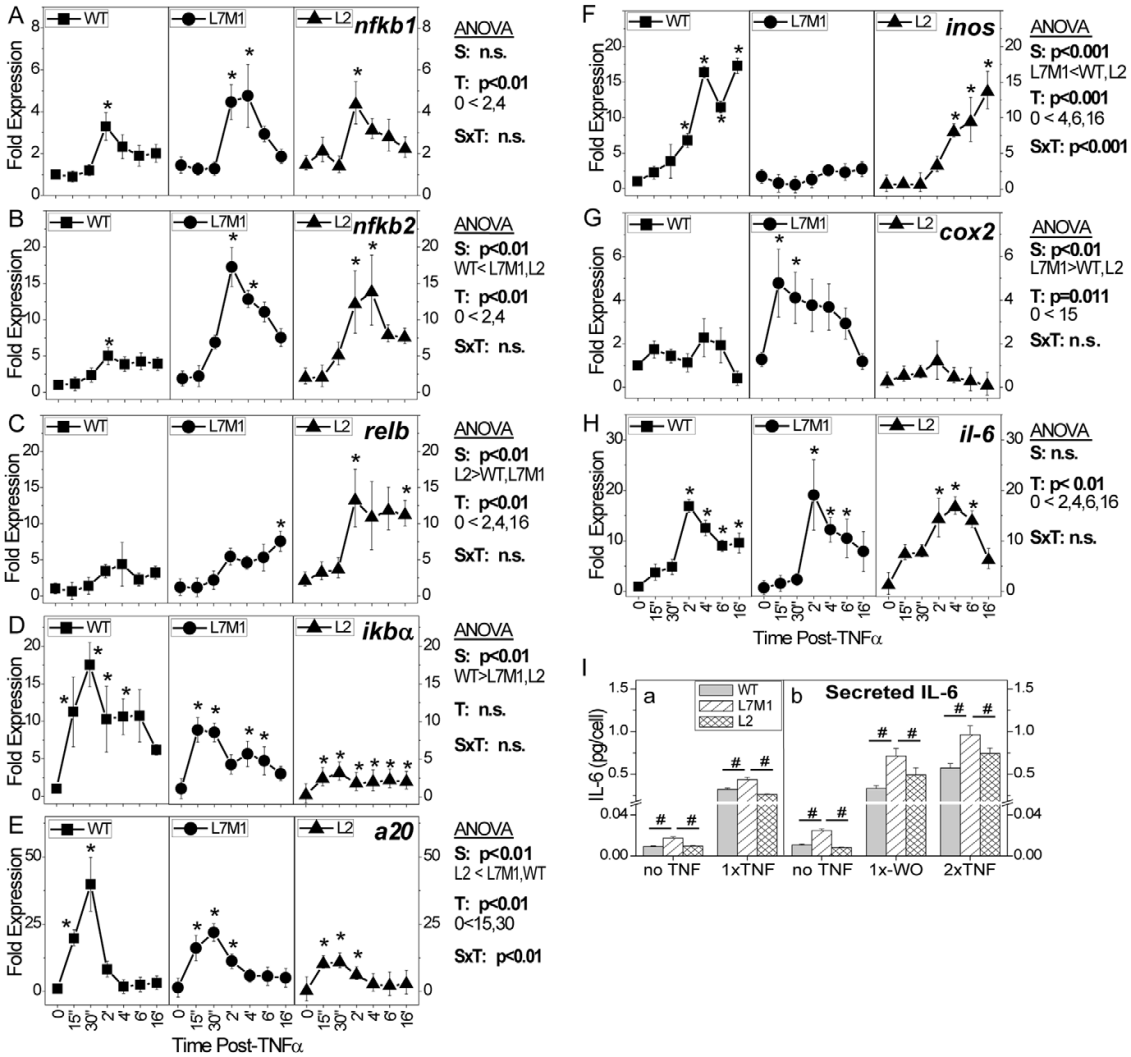
Expression of NFκB Responsive Genes Following TNFα Stimulation. Measures of gene expression were performed by quantitative RT-PCR for transcription factors *nfκb2* (p105) (**A**), *nfκb1* (p100) (**B**), *relB*(**C**), inhibitors of the NFκB pathway *iκbα* (**D**) and *a2 0*(**E**), and three prototypic responsive genes, inducible nitric oxide synthase (*inos*) (**F**), cyclooxygenase 2 (*cox2*) (**G**), and interleukin-6 (*il-6*) (**H**). Graph shows changes in expression relative to WT 0 (no TNFα) following stimulation of RPE cells from WT or KO (L7M1, L2) mice with TNFα (10 ng/ml). The response was normalized to ARBP for each sample. Data shown in A, D, G, and H are the mean (± SEM) of three independent experiments performed in triplicate. Data shown in B, C, E and F are the mean (± SEM) of two independent experiments performed in triplicate. Two-way ANOVA results are shown in each panel for (S) strain, (T) time post-TNFα, and (SxT) interaction. One-way ANOVA was performed for each strain over time to determine if there was a significant treatment effect. Results of post-hoc comparisons showing significant difference to no TNFα are indicated by * (p<0.05). (**I**) Secreted IL-6 was measured in culture media by ELISA. (a) IL-6 content was measured 48 hours after either a media change (no TNF) or a single dose of TNFα (1X TNF). (b) IL-6 content was measured after either a media change (no TNF), a single dose of TNFα followed by a media change (wash out) 48 hrs later (1X WO), or a single dose of TNFα followed by a second dose of TNFα (2X TNF) 48 hrs later. Results of one-way ANOVA comparing each cell line per treatment indicates significant differences by # (p<0.05). Data shown are the mean (± SEM) of three independent experiments run in triplicate.

In monitoring the time-dependent change in expression for *nfκb1* (p105), all cells exhibited a 3- to 4-fold increase in expression at 2 hours (Fig. 2A). For *nfκb2* (p100), a robust upregulation was also observed by 2 hrs in all cells (Fig. 2B). However, *nfκb2* expression in i-proteasome-deficient cells was 3-fold higher than in WT cells (p<0.01). For *relb*, expression was increased ∼five-fold by 2–4 hrs post treatment in WT and L7M1 cells (Fig. 2C). The response was significantly greater in L2 cells; a 10-fold increase in *relb* expression was observed from 2 to 16 hrs post-TNFα (p<0.01).

Expression was also monitored for the inhibitory proteins *iκba* and *a20*. For the early phase inhibitor IκBα, TNFα stimulated an 18-fold increase in expression in WT cells by 30 min, followed by a slight decrease in expression throughout the 16 hrs (Fig. 2D). This sustained, elevated expression reflects the chronic stimulation by TNFα. I-proteasome-deficient cells exhibited similar kinetics of upregulated expression, but of significantly lower magnitude. The maximum expression was only 10-fold and 3-fold over unstimulated values for L7M1 and L2 cells, respectively.

Expression of the late phase inhibitor *a20* was also robustly upregulated by 30 min post treatment, followed by a rapid decline to slightly above baseline levels in all cells (Fig. 2E). While the kinetics of the response was similar between cells, there were significant differences in the magnitude of response. Maximum levels of expression were 40-, 20-, and 12-fold over baseline values in WT, L7M1, and L2 cells, respectively (p<0.01).

Quantitative PCR of three prototypic NFκB responsive genes showed that in WT and L2 cells, *inos* was significantly upregulated by 2 to 4 hours, respectively (Fig. 2F). In contrast, L7M1 did not respond. For *cox2*, there was no response in WT and L2 cells, but a robust increase by 15 minutes post-TNFa treatment in L7M1 cells (Fig. 2G). Measures of *il6* expression showed that all cells exhibited a similar response; a significant ∼16-fold upregulation was observed by 2 hours (Fig. 2H). To explore potential differences downstream of expression, the content of IL-6 secreted into the culture medium was monitored. We found that the amount of IL-6 produced by L7M1 cells was significantly higher at baseline, at 48 hours after a single dose of TNFα, and at 96 hours after combinations of multiple treatments (Fig. 2I). These results show that while regulation of expression was approximately equal, there were differences in production and secretion of IL-6 that were influenced by the absence of LMP7 and MECL. In fact, the hyperactive response of L7M1 cells suggests potential problems in modulating the NFκB signal.

Results from quantitative PCR show that the absence of specific i-proteasome subunits altered the expression of multiple NFκB responsive genes. These results provide strong experimental evidence supporting a role for i-proteasome in regulating NFκB signaling.

### Altered NFκB transcription factor content and translocation

**Figure 3:**
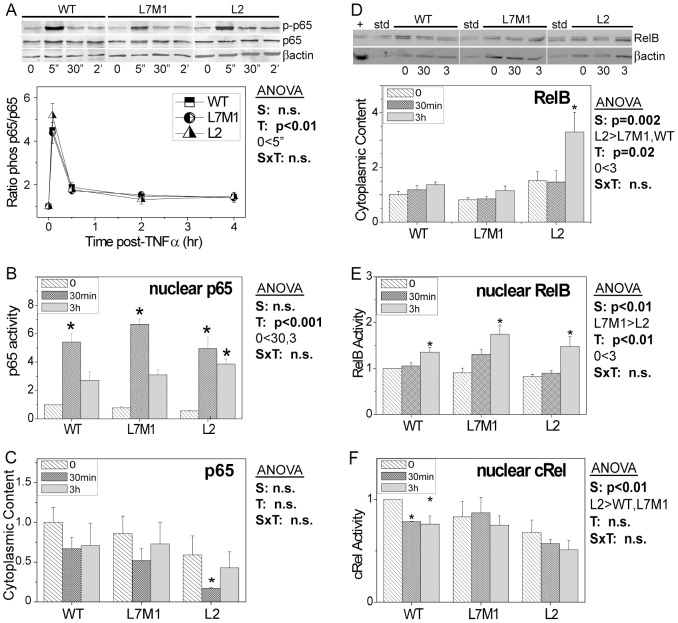
Activation and Protein Content for p65 (RelA), RelB, and cRel. (**A**) Western blots showing reactions for phosphorylated p65 (p-p65), p65,and βactin in cell homogenates from WT and i-proteasome-deficient (L7M1 and L2) cultured RPE cells at baseline (no TNFα) and from 5 min (5″) to 4 hrs with TNFα (10 ng/mL). Graph shows the ratio of phosphorylated to total p65 content before and after TNFα. *5 min is significantly higher than no TNFα for each cell type. Data are the mean (± SEM) of five independent experiments from n = 2 cell lines/group. (**B**) Transcription factor p65 activation was monitored in nuclear fractions using the Trans-Am NFκB transcription factor assay kit. Graphs show the mean (± SEM) of five independent experiments performed in duplicate from n = 2 cell lines/group. (**C**) Western blotting measured the content of p65 in cytoplasmic fractions from cells harvested before and after TNFα stimulation. Data are representative of three independent experiments. (**D**) Western blotting measured the content of RelB in cytoplasmic fractions from cells harvested before and after TNFα stimulation. βactin is the loading control. TNFα treated HeLa cells (+) were the positive control. Immune reactions were normalized to a standard sample (std) run on each blot. Protein loads were 10 µg per lane. Graphs show the mean (± SEM) of five independent experiments from n = 2 cell lines/group. (**E,F**) Transcription factor activation of RelB (E) and cRel (F) was monitored in nuclear fractions using the Trans-Am NFκB transcription factor assay kit. Data shown in E and F are the mean (± SEM) of three independent experiments performed in duplicate. Two-way ANOVA results are shown in each panel for (S) strain, (T) time post TNFα and (SxT) interaction. One-way ANOVA was performed for each strain over time to determine if there was a significant treatment effect. Results of post-hoc comparisons showing significant difference with no TNFα are indicated by * (p<0.05).

To investigate potential mechanisms responsible for the altered response to NFκB signaling in i-proteasome deficient cells, multiple assays were employed to monitor the phosphorylation state, protein content, and nuclear translocation of NFκB transcription factors. An early step in activation of p65 involves its phosphorylation following degradation of the inhibitor protein IκBα [33]. Western immunobloting was used to monitor both the phosphorylated p65 and the total p65 population (Fig. 3A). The data shows a rapid peak of phosphorylation within 5 minutes of TNFα treatment indicative of p65 activation in all cell lines. This rapid activation was followed by a decline in the ratio of phosphorylated to total p65 at 30 minutes that was maintained at just above baseline for at least 4 hours. The pattern of activation was replicated in the measures of p65 nuclear content; a significant 5- to 6-fold increase was observed for all cell lines at 30 min post-TNFα indicating robust translocation (Fig. 3B). By 3 hrs, p65 content was reduced approximately 50% in WT and L7M1 cells, but did not decrease in the L2 cells. Additionally, measures of the overall cytoplasmic content of p65 were not different between cells, although there was significantly less p65 in L2 cells at 30 min (Fig. 3C). These results suggest that p65 activation is not regulated by the i-proteasome, but there is evidence for delayed termination of p65 activation in cells deficient in LMP2.

RelB is part of the Alternative Pathway and a preferred partner of p52 [34]. In comparing the overall cytoplasmic content of RelB, L2 cells contained significantly higher levels than WT and L7M1 cells (p<0.01) (Fig. 3D). In all cells, treatment with TNFα resulted in a significant increase in the cytoplasmic content (p = 0.02) by 3 hrs post treatment. These results are consistent with the time dependent change in expression, which showed a significant increase in message by 2 hrs in all cells (Fig. 2C). Additionally, upregulation in L2 cells was greater than 2-fold more than both WT and L7M1, which is consistent with the higher RelB protein content. TNFα also induced nuclear translocation of RelB by 3 hrs, as suggested by the 40% (WT) to 80% (L7M1, L2) increase in RelB nuclear content (p<0.01) (Fig. 3E).

The nuclear content of cRel was also monitored before and after TNFα treatment (Fig. 3F). Overall, the nucleus of L2 cells contained significantly lower amounts of cRel compared with both WT and L7M1 (p<0.01). While there was a significant decrease in cRel content in WT cells after 30 min and 3 hr of TNFα stimulation (p = 0.014), nuclear cRel content did not change in L7M1 and L2 cells. Based on these results, showing minimal differences in cRel response, we did not pursue additional assays for this transcription factor.

**Figure 4.Content:**
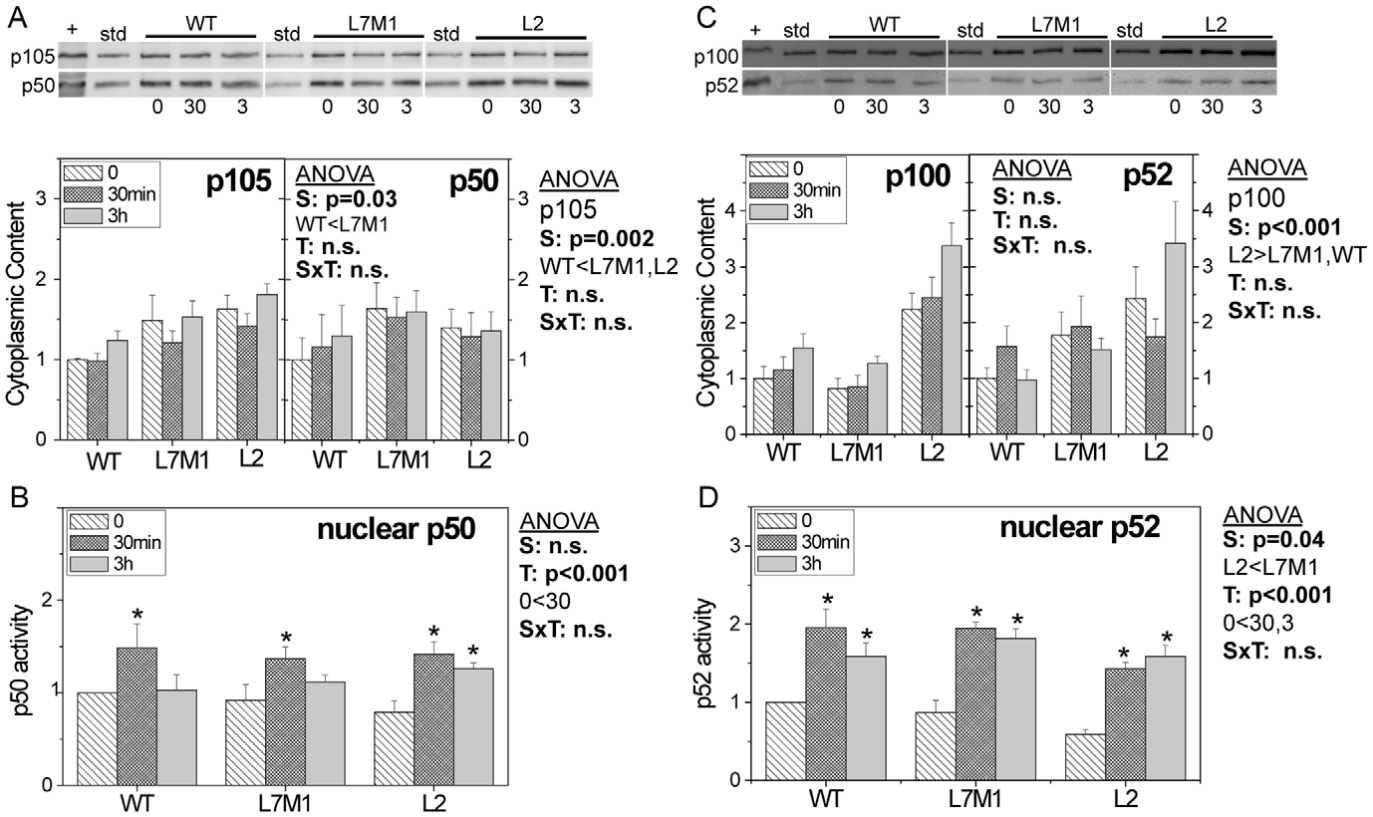
and activation of p105/p50 and p100/p52. (**A,C**) Protein content of transcription factor precursor p105 and its active form, p50 (A) and precursor p100 and its active form, p52, were measured from Western blots using cytoplasmic fractions from cells harvested without TNFα or after 30 min and 3 hrs of TNFα stimulation. TNFα treated HeLa cells (+) were used as a positive control. Immune reactions were normalized to a standard sample (std) run on each blot. Protein loads were 10 µg per lane. Graphs show the mean (± SEM) of six independent experiments from n = 2 cell lines/group. (**B,D**) Transcription factors p50 (B) and p52 (D) activation were monitored using the Trans-Am NFκB Transcription Factor assay kit and the nuclear fraction from cells harvested before and after TNFα stimulation. Graphs show the mean (± SEM) of five independent experiments done in duplicate. (Data are from n = 2 cell lines/group) Two-way ANOVA results are shown in each panel for (S) strain, (T) time post TNFα and (SxT) interaction. One-way ANOVA was performed for each strain over time to determine if there was a significant treatment effect. Results of post-hoc comparisons showing significant difference with no TNFα are indicated by * (p<0.05).

Comparing the content of p105, the precursor for the active transcription factor p50, significantly higher levels were observed in L7M1 and L2 compared with WT cells (p<0.01)(Fig. 4A). For p50, the overall content was higher in L7M1 cells, but did not vary with TNFα treatment in any cell line. There was also no cell-dependent difference in the overall content of p50 in the nucleus at baseline and at 30 minutes post-TNFα treatment (Fig. 4B), which was increased ∼50% at 30 min over basal levels in all cells. For WT and L7M1 cells, p50 levels returned to baseline by 3 hours. In contrast, cells deficient in LMP2 exhibited higher levels even at 3 hours, suggesting prolonged activation of p50. These data, along with the results showing elevated levels of nuclear p65 at 3 hrs (Fig. 3B) suggest that cells deficient in LMP2 have delayed termination of the Classical Pathway.

Protein content of the p100 precursor was significantly higher in L2 cells compared with WT and L7M1 cells (p<0.001). The p52 content was also 2 to 3-fold higher in L2 cells, although this increase was not statistically different (Fig. 4C). The higher overall content of p100 in L2 cells could be due to the 3-fold higher expression of the *nfκb2* gene in i-proteasome-deficient cells (Fig. 2B). TNFα also stimulated the translocation of p52 into the nucleus (Fig. 4D). At 30 min and 3 hrs post treatment, nuclear content was ∼2-fold higher than baseline levels (p<0.01). Of note, L2 had significantly less nuclear p52 compared with WT and L7M1 cells (p = 0.04). Considering the higher p100 present in the cytoplasm, the lower nuclear p52 suggests a defect in nuclear translocation of this transcription factor in cells deficient in LMP2.

### Altered content of NFκB Regulators

**Figure 5.Content:**
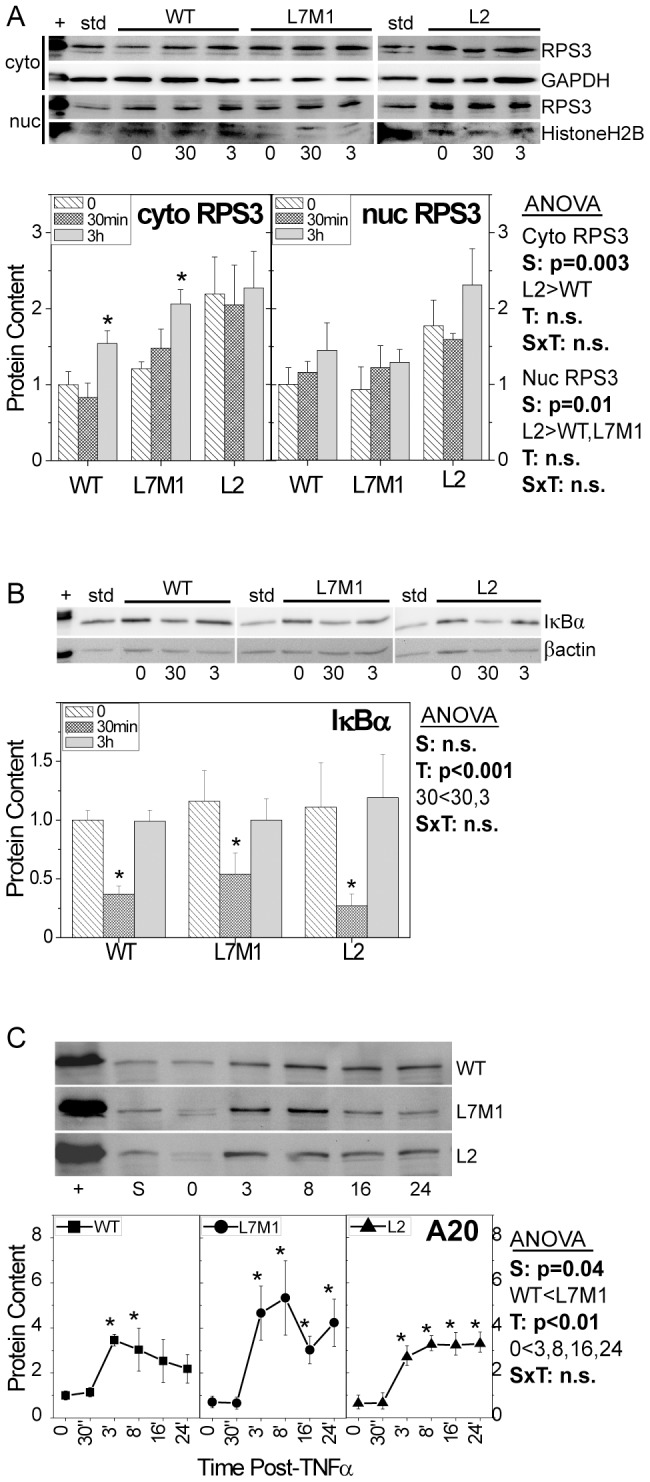
of Protein Regulators of NFκB Signaling. The cytoplasmic and nuclear content of the NFκB pathway enhancer Ribosomal Protein S3 (RPS3) (**A**) and the cytoplasmic content of inhibitory proteins IκBα(**B**) and A20 (**C**) were determined from Western blots for cells harvested without TNFα or after 30 min and 3 hrs of TNFα stimulation. Data shown in A and C are the mean (± SEM) of four independent experiments. Data shown in B are the mean (± SEM) of six independent experiments from n = 2 cell lines/group. TNFα treated HeLa cells (+) were used as a positive control. Immune reactions were normalized to a standard sample (std) run on each blot. Protein load was 10 µg per lane for Western blots in B and C. Protein load was 25 µg per lane for Western blots in A. Two-way ANOVA results are shown in each panel for (S) strain, (T) time post TNFα and (SxT) interaction. One-way ANOVA was performed for each strain over time to determine if there was a significant treatment effect. Results of post-hoc comparisons showing significant difference with no TNFα are indicated by * (p<0.05).

NFκB signaling is regulated by multiple accessory proteins that either enhance or inhibit expression of target genes. Ribosomal protein S3 (RPS3) is a multifunctional protein that enhances NFκB transactivation by stabilizing p65 binding to specific κB sites [35]. Measures of the cytoplasmic content showed significantly elevated levels of RPS3 in L2 compared with WT cells (Fig. 5A, left panel) (p<0.01). While TNFα had no effect on RPS3 cytoplasmic content in L2 cells, there was a significant increase in both WT and L7M1 cells at 3 hours. There was also no measurable change in nuclear RPS3 content in response to TNFα, although RPS3 content was higher in L2 compared with both WT and L7M1 cells (Fig. 5A, right panel). The elevated nuclear content of RPS3 in L2 cells may enhance binding of the p65/p50 dimer on the promoter. This mechanism could help explain the extended presence of p65 and p50 in the nucleus of L2 cells following TNFα stimulation.

The content of two inhibitors of NFκB signaling, IκBα and A20 was also examined. In monitoring IκBα protein content, basal levels were equivalent between cells (Fig. 5B). In response to TNFα stimulation, all cells displayed the expected initial decrease in IκBα content at 30 min, followed by a recovery to baseline levels by 3 hrs. This rapid rebound in IκBα content is due to the upregulated expression of the *iκBα* gene that occurred within 15 minutes in all cells during the early phase of signaling (Fig. 2D). Cells had similar basal content of A20 and exhibited a significant 3- to 5-fold increase by 3 hrs and remained elevated through 24 hrs post-TNFα treatment (Fig. 5C).

**Figure 6:**
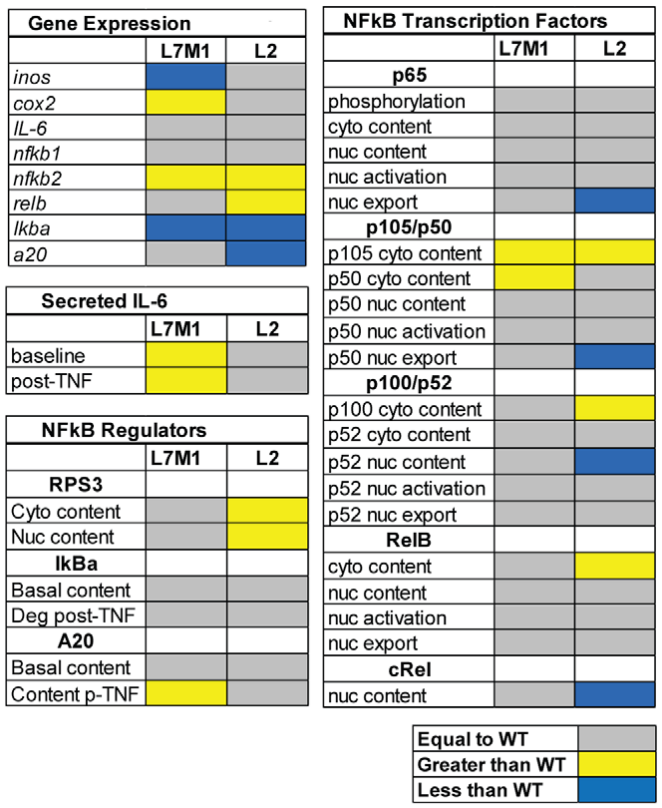
Summary of results for i-proteasome-deficient cells. Colored boxes summarize results of experiments for cells deficient in i-proteasome (L7M1 and L2) compared with WT cells. Grey indicates results were not different than WT. Yellow or blue boxes indicate results that were either greater or less than results obtained in WT cells. Cyto = cytoplasmic; deg = degradation; nuc = nuclear; p-TNF = post-TNFα.

Figure 6 provides a graphical summary of study results comparing the L7M1 and L2 cells with WT cells. Cells deficient in i-proteasome subunits exhibited a number of striking differences in basal content of NFκB proteins, as well as in their response to TNFα treatment. Additionally, there were substantial differences between cells lacking one (*lmp2^−/−^*) or two (*lmp7^−/−^*/*mecl-1^−/−^*) i-proteasome catalytic subunits.

## Discussion

In this study, we tested the hypothesis that the i-proteasome is involved in regulating NFκB signaling using RPE cells derived from WT mice and those deficient in one (L2) or two (L7M1) i-proteasome catalytic subunits. A single, sustained dose of TNFα was used to stimulate NFκB signaling. Downstream early- and late-phase events, such as activation and nuclear translocation of NFκB transcription factors, expression of NFκB responsive genes and content of regulatory proteins, were monitored to begin identifying specific sites in the NFκB pathway that are altered due to i-proteasome deficiency.

Results showed equivalent TNFα-induced activation of the Classical Pathway, including rapid p65 phosphorylation, degradation of the inhibitory protein IκBα, and translocation of p65 and its prototypic binding partner p50 into the nucleus (Fig. 6). These results suggest activation of the Classical Pathway is independent of i-proteasome regulation. Termination of the signal, as evidenced by decreased content of nuclear p65/p50 by 3 hr, was similar between WT and L7M1. In L2 cells, there was no reduction in nuclear content of these transcription factors, suggesting delayed termination of the Classical Pathway. This delay may be due to increased nuclear content of RPS3, a protein that stabilizes the p65/p50 dimer on the promoter. Cells lacking the LMP2 subunit demonstrated other substantial changes in transcription factor content and activation. There was decreased nuclear translocation of p52 despite having higher cytosolic content of the precursor p100. The lower nuclear p52 content could a result of p100 sequestering p52 in the cytosol and preventing its translocation. Notably, RelB expression and content were significantly higher in L2 cells. Since RelB and p52 are the prototypic binding partners in the Alternative Pathway, the reciprocal change in content for these two transcription factors suggest the composition of dimers that signal via the Alternative Pathway is altered. These alterations in transcription factor content and activation in L2 cells could help explain the significant difference in expression of NFκB responsive genes. For the L7M1 cells, cytoplasmic content of p105/p50 and the inhibitory protein A20 were elevated. Changes in these NFκB regulators and in regulation at points in the NFκB pathway outside of those monitored in this study, as well as the 2.5-fold increase in LMP2 content compared with WT cells, could contribute to the mechanism responsible for the altered expression of NFκB responsive genes and increased levels of secreted IL-6 at baseline and after TNFα treatment.

Our results showing that activation of the Classical Pathway is not affected by the absence of i-proteasome subunits is consistent with a recent study that used a chemical genetic approach [36]. The authors observed no defects in TNFα-induced activation of the Classical Pathway (i.e., degradation of IκBα, nuclear translocation of p65/p50) using small molecule inhibitors that specifically target either the LMP2 or LMP7 subunits. The Classical Pathway is the signaling pathway that is most consistently activated following many different stressors. The observation that this pathway was unaffected by i-proteasome- deficiency could explain why i-proteasome KO mice are viable and exhibit limited changes in processes associated with NFκB signaling.

Data from the current study show the most substantial differences in NFκB signaling occur in cells lacking the LMP2 subunit. Previous studies performed in T2 human lymphocyte cell lines, embryonic fibroblasts and spleen cells from LMP2 KO mice, and spleen tissue from non-obese diabetic mice also showed altered NFκB signaling. These model systems, which were deficient in the LMP2 subunit, showed defects in proteolytic processing of NFκB precursors (p100/p105) to the active transcription factors (p52/p50), and decreased degradation of IκBα after TNFα stimulation [18], [19]. In studies of B cells from LMP2-deficient mice, delayed and less complete degradation of IκBα following lipopolysaccharide stimulation was observed [20]. In contrast to these published reports, the present study showed L2 cells had higher overall levels of p100 and p105, but there was no evidence for delayed proteolytic processing of the precursors since the levels of the active transcription factors p50 and p52 were equivalent to WT cells. Runnels et al also reported that the presence of LMP2 was not obligatory in the processing of p105 based on the generation of mature p50 protein in T2 cells with the LMP2 gene deleted [22]. Potential explanations for the discrepancies between studies include cell- and tissue-specific differences in i-proteasome populations and their response to TNFα. Substantial cell-specific differences in subunit content was clearly demonstrated in the current study when comparing cultured RPE cells, RPE in vivo, and cerebellum, and in our previous studies comparing retina and spleen proteasomes in KO mice [8]. In cultured RPE, we observed a dramatic increase in LMP2 content in L7M1 cells, which could be a compensatory response to the loss of LMP7 and MECL that is compounded by the absence of an in vivo environment and regulation from neighboring tissue. This overcompensation in LMP2 content by L7M1 could help explain the differences in response to TNFα exhibited by the two i-proteasome-deficient cell lines.

The p100 precursor is constitutively processed to p52. In all cells, a steady level of precursor and processed protein is maintained, suggesting this is a tightly regulated and ongoing process. In L2 cells, the significantly higher levels of p100 could have a significant impact on gene transcription due to its multiple regulatory roles. For example, p100 sequesters both p65 and RelB in the cytoplasm, thus inhibiting both the Classical and Alternative pathways. Induced expression of p100 following TNFα stimulation facilitates the exchange of p65-containing dimers for p52/RelB, which is insensitive to negative feedback from IκBα and promotes late-phase transcription of genes [14]. It is also possible that the higher content of p100 in L2 cells is a compensatory response to higher levels of the RPS3 enhancer. Higher p100 may be required to keep NFκB activation in check.

In L7M1 cells, increased *cox2* expression, higher basal levels of secreted IL-6 protein, and sustained IL-6 production following TNFα treatment suggests cells lacking the LMP7 and MECL-1 subunits have a heightened inflammatory response. Our results are consistent with earlier reports of enhanced TNFα-induced production of IL-6 in cultured fibroblasts, tissues, and serum from patients with Nakajo-Nashimura Syndrome [37]. This syndrome is one of a spectrum of recently described auto-inflammatory diseases that are associated with mutations in the LMP7 protein and lower i-proteasome content [23]. Analysis of tissues and cells from patients harboring mutations in the *lmp7* gene has shown an accumulation of ubiquitinated and oxidized proteins that are likely due to the loss in i-proteasome function. The proposed mechanism responsible for the heightened inflammatory response included the loss of i-proteasome-dependent degradation of damaged proteins, which triggers hyperactivation of the p38 MAP kinase pathway and overproduction of the inflammatory cytokine IL-6 [23].

One of the most intriguing results from our study is that the absence of specific i-proteasome catalytic subunits produced a unique response in the NFκB pathway. For example, multiple genes were differentially affected by the absence of specific i-proteasome subunits. Potential mechanisms include differences in content of NFκB transcription factors, cross-talk between signaling pathways, and differences in NFκB regulation outside of the current study. There is a strong possibility that differences in composition of the active transcription factor dimers in L2 cells, where p100, p105, and RelB are found in greater abundance, plays a critical role in the differential regulation of NFκB genes. Studies in knock-out mice have suggested that each dimer carries out specific regulatory roles in transcription via selective protein-protein interactions with other transcription factors, co-regulators (i.e., RPS3), and chromatin proteins [38]. Also contributing to the dimer-specific response is their unique DNA-binding specificities and affinities for target genes. Recent evidence has also suggested that dimers can adopt specific conformations when bound to different DNA sequences, which may also influence the binding affinity and kinetics of transcription [39]. Post-translational modifications can also influence the location, stability and interactions with DNA and other transcription factors. These modifications are regulated by not only the NFκB family of proteins, but also other signaling pathways, demonstrating the importance of cross talk between pathways in regulating signaling.

While our study provided a limited examination of specific proteins, it is possible that differences in components upstream and downstream of our focus could also influence the outcomes measured. For example, macrophages isolated from mice lacking the LMP7 and MECL subunits had significantly reduced iNOS expression following lipopolysaccharide stimulation [40]. The difference in expression was due to defects in signaling from the toll-like receptor 4 via the TRIF/TRAM pathway, which are upstream of NFκB signaling. It is also possible that i-proteasome's effect is broader in scope and affects other pathways, including those that cross talk with NFκB. One pathway with suggested regulation by the i-proteasome is Protein Kinase B (Akt) signaling, which is controlled by the molecule phosphatase and tension homologue deleted on chromosome 10 (PTEN). This protein is an important regulator of cardiac muscle size and cellular changes associated with cardioprotection during ischemia preconditioning. Of note, PTEN content is regulated by the presence LMP2. As demonstrated in KO mice, the absence of LMP2 resulted in higher PTEN levels, significantly smaller hearts, cardiomyopathy, and insensitivity to ischemia preconditioning [41], [42]. The defects in cardiac function could be rescued in LMP2 KO heart muscle by treating with a PTEN inhibitor.

Additional reports provide compelling evidence for i-proteasome's role in regulating signaling in diverse biological processes. A recent study showed i-proteasome is involved in maintenance of pluripotency in human embryonic stem cells via degradation of proteins that regulate the cell cycle and control differentiation [43]. L7M1 KO mice and humans with LMP7 mutations both exhibit lipodystrophies that stem from defects in adipocyte maturation [23]. I-proteasome deficiency also had an adverse effect on visual function, suggesting defects in retinal signaling in mice lacking one (LMP7) or two (LMP7, MECL) i-proteasome subunits [44]. In L7M1 mice, we have also documented defects in corneal wound healing, a process involving both p38 MAP kinase and NFκB signaling [45].

Several mechanisms have been proposed to explain how the presence of specific proteasome subtypes (i-proteasome, standard and intermediate proteasomes) could differentially affect biological processes. Since the catalytic activity (i.e., degradation rate and substrate specificity) of each proteasome subtype differs, the repertoire of peptides produced will depend on the cell's proteasome population. Some of these peptides could be biologically active and regulate cell processes. For example, in a study examining the production of a specific peptide that was recognized by cytotoxic T lymphocytes, the authors reported that the peptide was generated by the i-proteasome and destroyed by the standard proteasome due to the presence of an acidic amino acid within the peptide's sequence [46]. Preservation of the peptide by the i-proteasome was explained by the exchange of β1 for LMP2, which leads to reduced cleavage of proteins after acidic amino acids. It is also possible that some intermediate proteasomes containing a mixture of different subunits could either enhance or compromise cell function, as shown previously for immune cells of LMP2 KO mice [47]. Decreased lymphocyte survival and reduced cytokine production, defects associated with impaired NFκB signaling, were attributed to the intermediate proteasomes contained in KO cells. The presence of different catalytic subunits could also change the conformation of the catalytic chamber, as suggested by the crystal structure of proteasome subtypes [48], and alter the specificity for substrates. Different catalytic subunits could also provide binding sites for protein and non-protein regulatory molecules, i.e., fatty acids, which could affect the kinetics of degradation. These ideas are supported by studies that reported different results when inhibiting subunit activity versus genetically eliminating the subunit [49], [50].

Another mechanism that could help explain i-proteasome's effect on diverse biological processes includes its enhanced ability to degrade oxidized, misfolded, and polyubiquitinated proteins. This property is particularly relevant in responding to conditions of stress and injury, which often involves oxidative stress and the subsequent production of damaged proteins. Considerable evidence has accrued in support of this function for i-proteasome. For example, oxidized and ubiquitin-marked proteins accumulate in cells and tissues deficient in i-proteasome with stress, aging, or disease [37], [50]–[53]. Conversely, cells overexpressing i-proteasome regulatory complex PA28 exhibit enhanced degradation of oxidized and misfolded proteins [54], [55]. Thus, the presence of i-proteasome provides protection from the proteotoxic accumulation of damaged proteins.

In summary, results from this study show substantial differences in regulation of the NFκB pathway that were unique to cells deficient in either the LMP2 or the LMP7 and MECL catalytic subunits of the i-proteasome. As a caveat, our results may be specific to cultured RPE cells and therefore, studies in different cells from KO mice are essential to determine the applicability of these results. Never the less, these data provide unequivocal evidence that i-proteasome performs functions that go beyond its role in generating peptides for antigen presentation and includes a central role in modulating NFκB signaling. Regulation may occur through degradation of proteins directly involved in the signaling cascade, or by an indirect mechanism via degradation of regulatory molecules or modulators of other pathways that crosstalk with NFκB. The presence of multiple signaling pathways that coordinate to convey compensatory effects help ensure cell viability is maintained. This strategy is likely to apply to the functions performed by the i-proteasome since mice with genetic ablation of all three i-proteasome subunits are viable [50]. It is possible that i-proteasome plays a role in fine-tuning the rate of signaling or downstream events of not only NFκB, but other signaling pathways. Considering that NFκB is the main pathway for responding to stress, our results firmly establish the i-proteasome at the center of the response to stress and injury.

## Supporting Information

Figure S1 **Overview of NFκB signaling following TNFα stimulation.** (**A**) In the Classical Pathway of NFκB activation, TNFα binding to TNF receptor (TNFR) triggers receptor trimerization and recruitment of adaptor proteins such as RIP1 (receptor interacting protein) to the intercellular domain of TNFR. RIP1 is modified by K63-linked ubiquitin and contributes to the recruitment, phosphorylation, and activation of the IkK complex (γ, α, β). Ikk phosphorylates the inhibitory protein IκBα, leading to ubiquitination and subsequent degradation by the proteasome.Proteolysis of IκBα releases the p50/p65 dimer and allows it to bind to the facilitator protein Ribosomal Protein S3 (RPS3). Following nuclear translocation, RPS3 enhances p65 binding on the NFκB promoter of target genes, including inos, cox2, il-6, iκbα, a20, nfκb1, nfκb2, and relb. The Alternative Pathway involves activation of IκKα, which phosphorylates p100 and p105 and initiates degradation of the inhibitory portion of p100 and p105 to generate p52 and p50, respectively. The prototypic binding partner of p52 is RelB.p50 can form heterodimers with p65 and take part in the Classical Pathway. Alternatively, p50/p50 homodimers can form, translocate into the nucleus and inhibit transcription. (**B**) Negative feedback includes transcription of several regulatory proteins that terminate NFκB signaling. Newly synthesized IκBα facilitates removal of p65/p50 from the nucleus and sequesters this dimer in the cytoplasm. Production of p100 also inhibits p65/p50 nuclear translocation. Expression of *a20* produces a dual functional enzyme that replaces K63-linked ubiquitin with K48-linked ubiquitin on RIP1, which target it for proteasomal degradation. A loss of RIP1 ends NFκB activation.
(TIF)

Figure S2 **Proteasome content in Cerebellum.** Western blots showing reactions for proteasome subunits in cerebellar homogenates harvested from WT and i-proteasome deficient mice (L7M1 and L2). Proteins loads were 5 µg per lane for α7, β1, and β5. Protein loads were 35 µg per lane for LMP2 and LMP7. Glyceraldehyde 3-phosphate dehydrogenase (GAPDH) was used as a loading control. The 20 s reaction was used as a positive control. Graph summarizes proteasome content in the cerebellum of WT and i-proteasome deficient mice (n = 3/group). One-way ANOVA results, *p = 0.01.
(TIF)
