# Supplementary figures and images for: Immunoproteasome Deficiency Modifies the Alternative Pathway of NFκB Signaling
Source: PLoS One. 2013 Feb 14;8(2):e56187. doi: 10.1371/journal.pone.0056187 (PMC3572990; doi:10.1371/journal.pone.0056187)

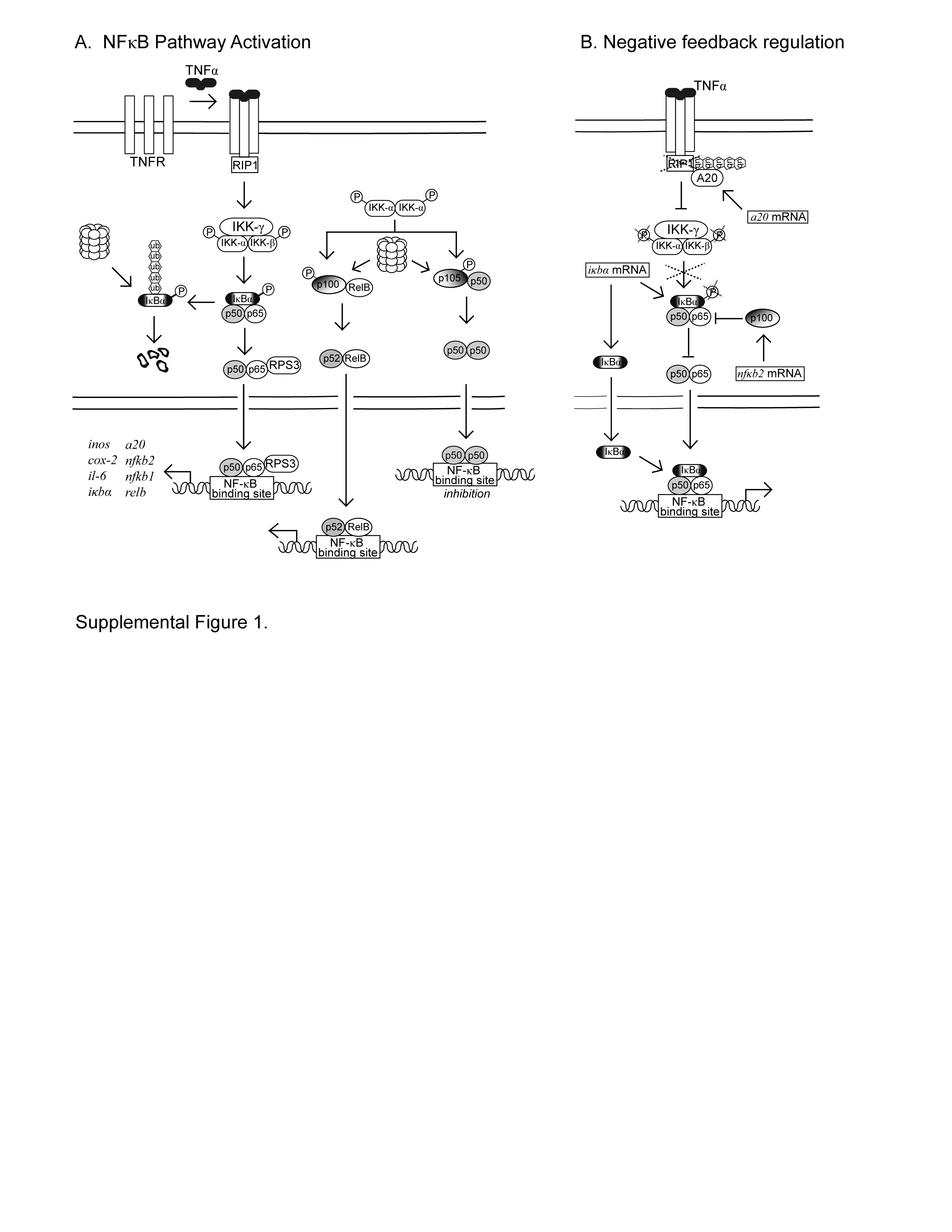

Supplement: Figure S1 — Overview of NFκB signaling following TNFα stimulation. (A) In the Classical Pathway of NFκB activation, TNFα binding to TNF receptor (TNFR) triggers receptor trimerization and recruitment of adaptor proteins such as RIP1 (receptor interacting protein) to the intercellular domain of TNFR. RIP1 is modified by K63-linked ubiquitin and contributes to the recruitment, phosphorylation, and activation of the IkK complex (γ, α, β). Ikk phosphorylates the inhibitory protein IκBα, leading to ubiquitination and subsequent degradation by the proteasome.Proteolysis of IκBα releases the p50/p65 dimer and allows it to bind to the facilitator protein Ribosomal Protein S3 (RPS3). Following nuclear translocation, RPS3 enhances p65 binding on the NFκB promoter of target genes, including inos, cox2, il-6, iκbα, a20, nfκb1, nfκb2, and relb. The Alternative Pathway involves activation of IκKα, which phosphorylates p100 and p105 and initiates degradation of the inhibitory portion of p100 and p105 to generate p52 and p50, respectively. The prototypic binding partner of p52 is RelB.p50 can form heterodimers with p65 and take part in the Classical Pathway. Alternatively, p50/p50 homodimers can form, translocate into the nucleus and inhibit transcription. (B) Negative feedback includes transcription of several regulatory proteins that terminate NFκB signaling. Newly synthesized IκBα facilitates removal of p65/p50 from the nucleus and sequesters this dimer in the cytoplasm. Production of p100 also inhibits p65/p50 nuclear translocation. Expression of a20 produces a dual functional enzyme that replaces K63-linked ubiquitin with K48-linked ubiquitin on RIP1, which target it for proteasomal degradation. A loss of RIP1 ends NFκB activation. (TIF) [file pone.0056187.s001.tif]

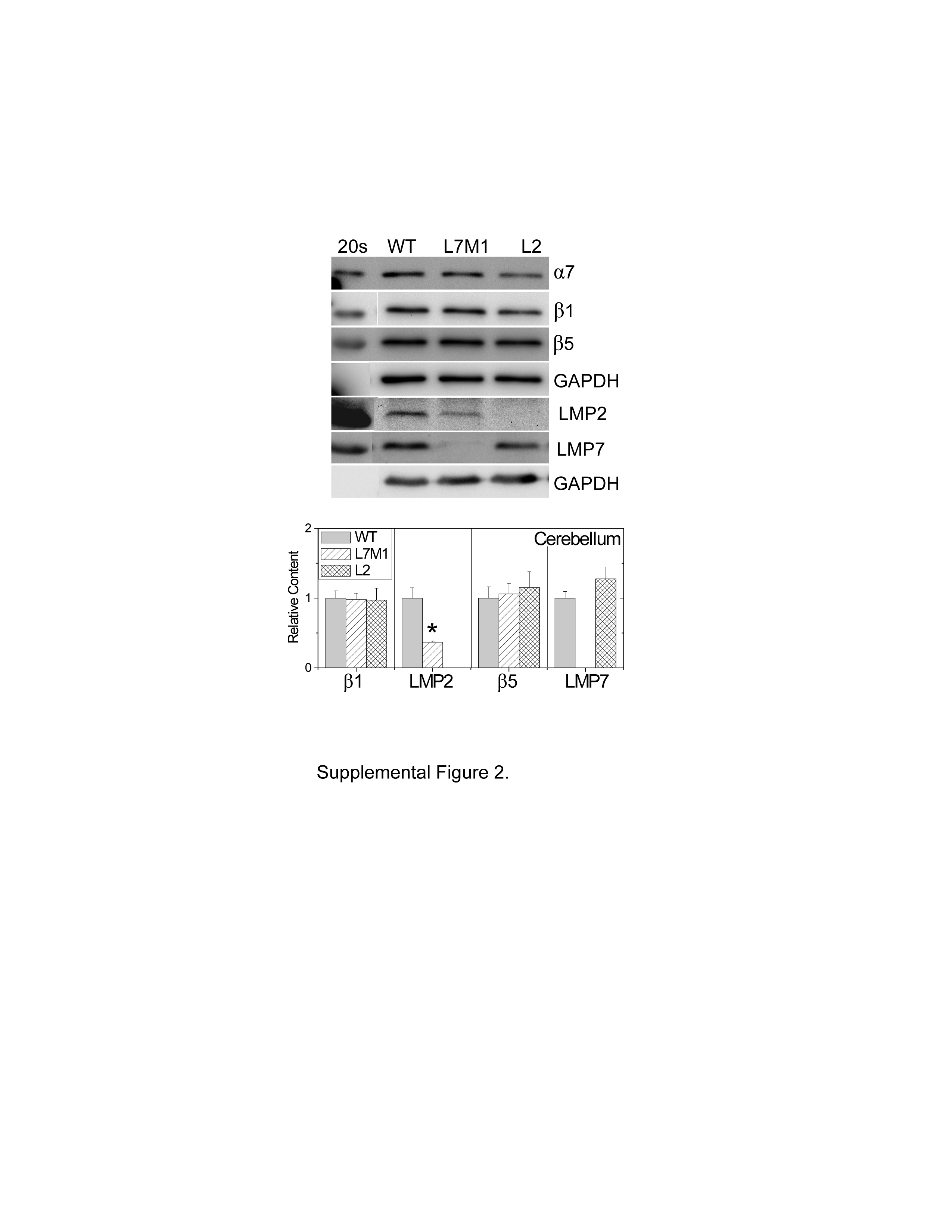

Supplement: Figure S2 — Proteasome content in Cerebellum. Western blots showing reactions for proteasome subunits in cerebellar homogenates harvested from WT and i-proteasome deficient mice (L7M1 and L2). Proteins loads were 5 µg per lane for α7, β1, and β5. Protein loads were 35 µg per lane for LMP2 and LMP7. Glyceraldehyde 3-phosphate dehydrogenase (GAPDH) was used as a loading control. The 20 s reaction was used as a positive control. Graph summarizes proteasome content in the cerebellum of WT and i-proteasome deficient mice (n = 3/group). One-way ANOVA results, *p = 0.01. (TIF) [file pone.0056187.s002.tif]
